# Who thinks what about e‐cigarette regulation? A content analysis of UK newspapers

**DOI:** 10.1111/add.13320

**Published:** 2016-03-11

**Authors:** Chris Patterson, Shona Hilton, Heide Weishaar

**Affiliations:** ^1^ MRC/CSO Social and Public Health Sciences UnitUniversity of GlasgowGlasgowUK

**Keywords:** Advocacy, e‐cigarettes, media, policy, regulation

## Abstract

**Aims:**

To establish how frequently different types of stakeholders were cited in the UK media debate about e‐cigarette regulation, their stances towards different forms of e‐cigarette regulation, and what rationales they employed in justifying those stances.

**Methods:**

Quantitative and qualitative content analyses of 104 articles about e‐cigarette regulation published in eight UK and three Scottish national newspapers between 1 January 2013 and 31 December 2014.

**Results:**

Reporting on e‐cigarette regulation grew significantly (*P* < 0.001) throughout the sample period. Governments and regulatory bodies were the most frequently cited stakeholders and uniformly supported regulation, while other stakeholders did not always support regulation. Arguments for e‐cigarette regulation greatly outnumbered arguments against regulation. Regulating purchasing age, restricting marketing and regulating e‐cigarettes as medicine were broadly supported, while stakeholders disagreed about prohibiting e‐cigarette use in enclosed public spaces. In rationalizing their stances, supporters of regulation cited child protection and concerns about the safety of e‐cigarette products, while opponents highlighted the potential of e‐cigarettes in tobacco cessation and questioned the evidence base associating e‐cigarette use with health harms.

**Conclusions:**

In the UK between 2013 and 2014, governments and tobacco control advocates frequently commented on e‐cigarettes in UK‐wide and Scottish national newspapers. Almost all commentators supported e‐cigarette regulation, but there was disagreement about whether e‐cigarette use should be allowed in enclosed public spaces. This appeared to be linked to whether commentators emphasized the harms of vapour and concerns about renormalizing smoking or emphasized the role of e‐cigarettes as a smoking cessation aid.

## Introduction

Electronic cigarettes (e‐cigarettes) have become increasingly popular in recent years, 1, 2 and are broadly considered to be less harmful than tobacco. However, the specific risks of e‐cigarette use and exposure are uncertain 3, and e‐cigarette regulation is hotly debated 4, 5. Some regard e‐cigarettes as useful tobacco cessation tools 6, 7, but concerns about negative impacts persist 8, 9. In addition to direct harms, fears exist that e‐cigarettes may reverse progress in de‐normalizing smoking 10 and stimulate tobacco use, particularly among young people 11.

In the United Kingdom, e‐cigarette products containing less than 20 mg of nicotine will be subject to various restrictions under the revised European Union Tobacco Products Directive (EU TPD) 12 from May 2016, while those containing more than 20 mg, or making medical claims, will need to be licensed as medicines by the Medicines and Healthcare Products Regulatory Agency to be sold. The Scottish Government, UK Department of Health and Welsh Government intend to introduce age‐of‐sale restrictions and bans on proxy purchasing 13, 14. Additionally, the Scottish Government plans to introduce restrictions on domestic e‐cigarette marketing, a register of e‐cigarette retailers, an age‐verification policy, a requirement to formally authorize under‐18s to sell e‐cigarettes and restrictions on domestic e‐cigarette marketing 13, 15.

E‐cigarettes have attracted media attention 16. Policymakers can be influenced by mass media coverage of public interest stories 17 as well as public opinion 18, and media content can demonstrably influence public understandings and opinions 19, 20, 21, 22, 23. Stakeholders can attempt to exert influence over public and political attitudes by engaging with media coverage of policy debates 24. Therefore, studying media debates about policy can increase understanding of how stakeholders and their positions are represented, which can help to inform advocacy in future debates in the United Kingdom and elsewhere. This content analysis study examines how stakeholders' positions on e‐cigarette regulation were represented in 2 years of UK newsprint media coverage.

## Methods

A time‐period of 1 January 2013 to 31 December 2014 was chosen to include the publication of the Scottish Government's Tobacco Control Strategy 25 in March 2013 and the commencement of consultations on e‐cigarette regulation by the UK, Welsh 26 and Scottish Governments in late 2014 27. The purposive sampling frame included eight UK and three Scottish national newspapers from the tabloid, middle‐market tabloid and quality genres 28, 29, 30 to ensure that a diverse range of readership profiles was represented 31. Each publication's Sunday counterpart was included, excluding the *Sun on* *Sunday*, which is not archived in the Nexis database.

The Nexis database was searched for articles containing three or more hits for the search term ‘e‐cig OR (electronic AND cigarette!) OR vape! OR vaping’, returning 738 articles. Each article was read and 634 were excluded on the basis of: not mentioning e‐cigarette regulation; being published in the television review, sports, travel, weather or readers' letters sections of newspapers; or being a duplicate. Following filtering, the sample comprised 104 articles.

The analytical aims were to establish: (1) how frequently different stakeholder categories were cited; (2) how frequently different stakeholder categories were associated with support for, or opposition to, different forms of e‐cigarette regulation; (3) what rationales were used to justify arguments about e‐cigarette regulation, and how frequently; and (4) which specific regulatory positions those corresponded with. Quantitative analysis was used to address the first three aims, while thematic qualitative analysis 32 was used to address the fourth. Each article was double‐coded by H.W. and C.P. Researchers coded over‐arching themes based on the codes assigned to the data, discussing differences in coding and interpretation of themes to reach consensus. Themes included the regulation of minimum age of purchase, marketing, e‐cigarette use in enclosed public spaces and e‐cigarettes as medicines.

To collect quantitative data, citations (either direct quotations or indirect mentions) of stakeholders were recorded. Tallies were kept of: how frequently each stakeholder was cited; how frequently they were presented as supporting or opposing regulation in general; how frequently they were presented as supporting or opposing each specific regulatory measure; and how frequently they were associated with specific rationales for their arguments. A multi‐level regression model was used to examine the rate of publication per quarter. To chart the frequency of citations of stakeholder groups against their stances towards regulation, an index was developed to reflect how consistently each stakeholder category was associated with support for regulation. The index expresses the proportion of all positive and negative arguments associated with a stakeholder that were in favour of regulation as a value on a linear scale from −1 (0% supportive) to 1 (100% supportive).

## Results

### Sample overview

The sample publications published 104 articles covering e‐cigarette regulation in 2013 (*n* = 28) and 2014 (*n* = 76), representing a mean of 4.7 articles per publication, per year (Supporting information, Table S1). Fifty‐five were published in quality genre publications, 28 in tabloids and 21 in middle‐market tabloids. Three‐quarters were published in UK publications (*n* = 76). A multi‐level regression model indicated that the rate of publication per quarter increased over time (*P* < 0.001), with a peak of 33 articles in Q4 2014 (Supporting information, Figure S1).

### Stakeholder categories

Stakeholders were categorized by organizational affiliation (Table 1). The most frequently cited groups were governmental and regulatory bodies (*n* = 50), politicians (*n* = 20), health charities (*n* = 18) and the e‐cigarette industry (*n* = 15). The most frequently cited individual stakeholders were the World Health Organization (WHO, *n* = 14), Scottish Government (*n* = 12), UK Government (*n* = 10) and the health charities Action on Smoking and Health (ASH) (*n* = 8) and ASH Scotland (*n* = 8).

**Table 1:** Frequency of arguments for specific regulatory measures by stakeholder category.

Type of regulation	*Government & regulatory bodies*	*Bodies representing health professionals*	*Health charities*	*Politicians*	*E‐cigarette industry*	*Academics*	*Total*
Regulation of minimum purchasing age	11	4	4	2	4	0	25
Regulation of marketing, advertising and promotion	16	3	3	1	0	1	24
Prohibition of e‐cigarettes in enclosed public spaces	11	4	1	1	0	0	17
Regulation of e‐cigarettes as medicine	11	1	2	1	1	1	17
Prohibition of proxy purchasing	1	0	0	0	0	0	1
Total	50	12	10	5	5	2	84

**Figure 1:**
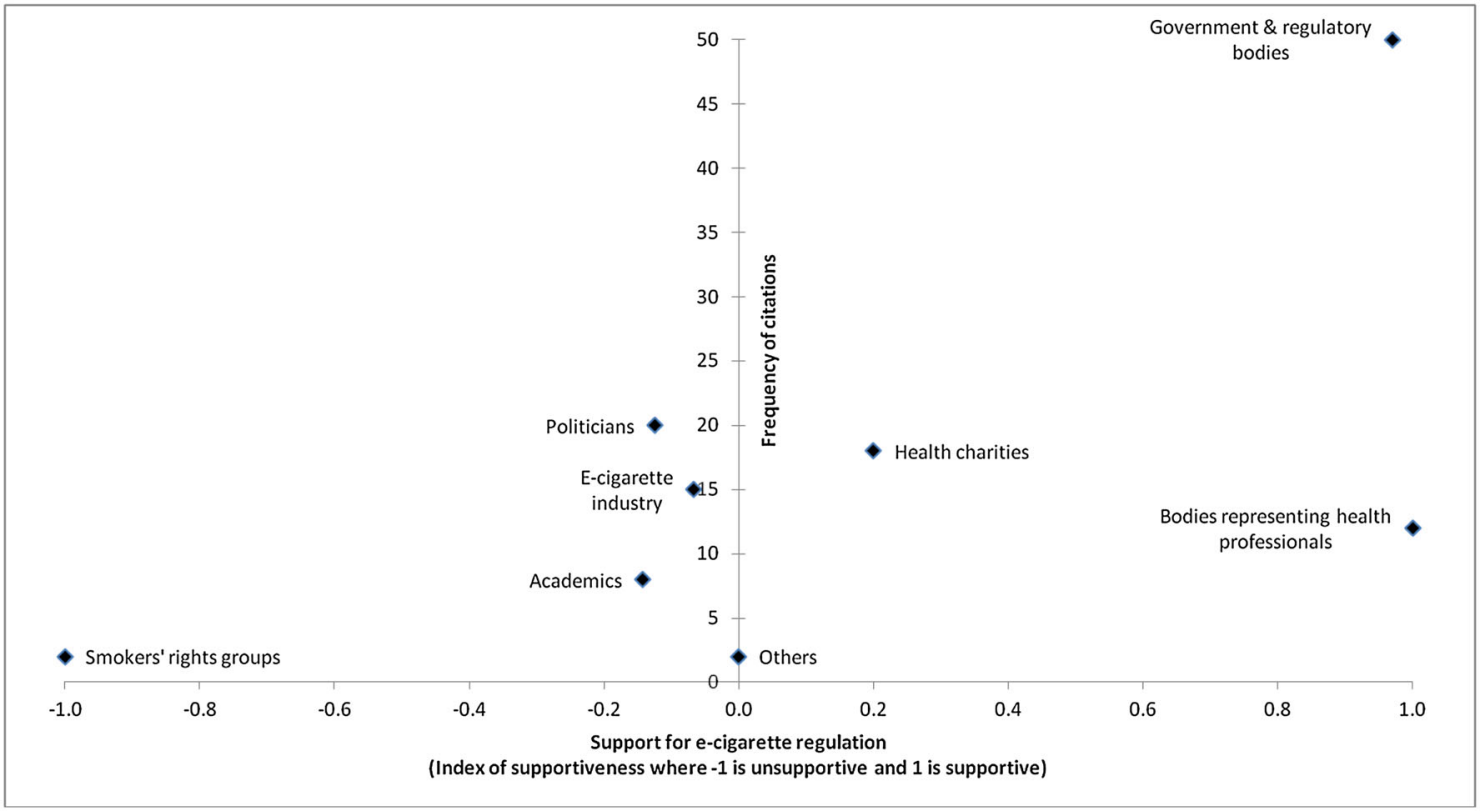
Frequency of citations of stakeholder categories and their aggregate stance towards regulation (*n* = 104)

Stakeholders were distributed along a continuum ranging from strong support for, and strong opposition to, e‐cigarette regulation (Fig. 1). Governments, regulatory bodies and bodies representing health professionals almost uniformly supported regulation, while the smokers' rights group FOREST (Freedom Organization for the Right to Enjoy Smoking Tobacco) consistently opposed regulation, albeit in just two citations. Politicians, health charities, manufacturers of e‐cigarettes and academics were associated with a range of arguments for and against e‐cigarette regulation.

E‐cigarette industry sources comprised independent companies and the Electronic Cigarette Industry Trade Association (ECITA), which represents 23 e‐cigarette brands 33. E‐lites, Socialites, Vapestick and Totally Wicked were associated with opposition to regulation, whereas JAC Vapour and Skycig were associated with support. ECITA was associated with equal proportions of arguments for and against regulation. During the sample period, ECITA did not represent any brands owned by transnational tobacco companies (TTCs), and the only TTC‐owned brand cited was Skycig (then owned by RJ Reynolds American, currently owned by Imperial Tobacco under the name Blu), which was cited as supporting age restrictions and regulating e‐cigarettes as medicine.

Political parties' stances towards regulation corresponded predominantly with their political alignment, consistent with Morley's 34 observation that pro‐tobacco forces tend to align with the political right. Labour Party, Green Party and Scottish National Party representatives were presented consistently as supportive of regulation, while Conservative Party, Liberal Democrats and UK Independence Party (UKIP) representatives were presented predominantly as opposed.

### Stakeholders' arguments about e‐cigarette regulation

Governmental and regulatory bodies (most frequently the WHO, Scottish Government and UK Government), health charities (primarily ASH and ASH Scotland) and bodies representing health professionals [primarily the British Medical Association (BMA)] tended to support the introduction of some form of regulation. Three‐quarters (*n* = 111) of the 146 arguments about e‐cigarette regulation attributed to stakeholders were in favour of regulation. Tables 1 and 2 detail the frequency of support for, and opposition to, specific types of regulation.

**Table 2:** Frequency of arguments against specific regulatory measures by stakeholder category.

	*Health charities*	*E‐cigarette industry*	*Smokers' rights groups*	*Total*
Type of regulation				
Prohibition of e‐cigarettes in enclosed public spaces	4	2	1	7
Regulation of e‐cigarettes as medicine	0	2	0	2
Regulation of marketing, advertising and promotion	0	0	1	1
Total	4	4	2	10

Fifty‐two arguments about e‐cigarette regulation were not related to any specific measure. Specific measures that stakeholders commented on included regulating: minimum purchasing age (*n* = 25); marketing, advertising and promotion (*n* = 25); the use of e‐cigarettes in enclosed public spaces (*n* = 24); and e‐cigarettes as medicines (*n* = 19). Of the 94 statements associated with specific measures, 84 (90.4%) were supportive and 10 (10.6%) opposed. Supporting information, Tables [Link], [Link], detail the frequencies of stakeholder categories' uses of different rationales.

A minimum age for purchasing e‐cigarettes was the most frequently supported measure (*n* = 25), associated frequently with child protection (*n* = 13). Tom Rolfe of Skycig welcomed ‘any regulations which will help us to ensure that under‐18s cannot access electronic cigarettes’ (*Scotsman*, 8 October 2013). Age‐of‐sale restrictions were justified by framing e‐cigarette use as a gateway to tobacco use (*n* = 5); Mark Drakeford of the Welsh Government highlighted the risk of ‘a new generation becoming addicted to [nicotine]’ (*Daily Mail*, 3 April 2014).

Marketing regulations were supported frequently (*n* = 24), and only opposed by Simon Clark of FOREST, who argued that ‘e‐cigarettes are increasingly popular with smokers who are trying to cut down or quit [...] and introducing greater restrictions on advertising, could do far more harm than good’ (*Scotsman*, 27 June 2014). Protecting children and young people was mentioned frequently (*n* = 20) in statements advocating marketing regulation; Deborah Arnott of ASH warned of children being ‘targeted’ by e‐cigarette marketing (*Guardian*, 28 April 2014).

Regulating e‐cigarettes as medicine was advocated 17 times and only opposed overtly twice, by ECITA. Support was rationalized by the need to ensure product safety and the potential of e‐cigarettes in tobacco cessation. Health concerns related predominantly to toxicity of e‐cigarette vapour, as well as risks associated with malfunctioning e‐cigarettes and the ingestion of e‐cigarette liquid. Kevin Fenton of Public Health England described the measure as essential to ‘assure people of their safety’ (*Daily Telegraph*, 26 November 2014). Deborah Arnott argued that the measure would ‘ensure [e‐cigarettes] are good quality [...] so they can be made available on prescription’ (*Observer*, 25 May 2014). Similarly, Dame Sally Davies, England's Chief Medical Officer, suggested that if the e‐cigarette vapour content were to be controlled, e‐cigarettes ‘might play a useful role in stopping smoking’ (*Daily Mail*, 3 April 2014). Criticism of regulating e‐cigarettes as medicine highlighted the burden on small e‐cigarette manufacturers and the comparative benefits to TTCs. Katherine Devlin of ECITA warned that regulation might ‘close out all the competition […so TTCs…] could get the whole market share for themselves’ (*Daily Mail*, 20 May 2013).

Prohibiting e‐cigarette use in enclosed public spaces was argued for 17 times and opposed seven times. The measure lacked the broad‐ranging support across stakeholder categories that other measures received, and was thus the key area of disagreement between public health stakeholders. Support for the measure was rationalized by citing: the risks associated with exposure to second‐hand vapour; the importance of protecting children; and the risk of re‐normalizing smoking. The WHO questioned the safety of second‐hand e‐cigarette vapour: ‘the fact [e‐cigarette] exhaled aerosol contains on average lower levels of toxicants than the emissions from combusted tobacco does not mean these levels are acceptable to involuntarily exposed bystanders’ (*Sunday* *Herald*, 31 August 2014). Dame Sally Davies cautioned against ‘normalising e‐cigarettes’ and ‘making smoking seem like a normal activity’ (*Daily Mail*, 19 May 2014). Dr Ram Moorthy of the BMA warned against reversing progress made towards making smoking ‘socially unacceptable’ (*Sun*, 22 May 2014).

Rationales used to oppose prohibiting e‐cigarettes in enclosed public spaces included their role in tobacco cessation and the limited evidence of risks. Tom Pruen of ECITA argued that ‘being able to use [e‐cigarettes] indoors is a big incentive for people to move away from tobacco’ (*Sunday* *Herald*, 31 August, 2014). Highlighting the limited evidence base, Neil McKeganey of the Centre for Drug Misuse Research described second‐hand exposure fears as ‘theoretical’ (*Sunday* *Herald*, 31 August 2014), while Hazel Cheeseman of ASH characterized ‘evidence of any harm to bystanders from use of these devices’ (*Herald*, 20 August 2014) as absent.

Incomplete evidence of the health risks of e‐cigarettes was cited by stakeholders from a range of categories, and used to support arguments both for and against regulation, indicating that different stakeholders used the inconclusive evidence base differently. Promoting a precautionary approach, John Middleton of the Faculty of Public Health stated that ‘We don't yet have enough evidence yet [*sic*] of the impact [e‐cigarettes] are having on other people’ (*Daily Mail*, 17 December 2014). Conversely, Charlie Hamshaw‐Thomas of E‐Lites, advocating a harm‐reduction approach, characterized the BMA as ‘“experts” without evidence playing puppet to the pharmaceutical industry's agenda’ (*Scotland* *on* *Sunday*, 20 October 2013).

## Discussion

We examined UK newsprint representations of the growing e‐cigarette regulation debate, highlighting the stakeholders involved and their stances towards regulation. The research findings are subject to certain limitations. As with most media analyses, the extent to which stakeholders' actual positions were distorted in media representations is unknown. However, given the impact of media representations on public understandings, media representations are no less important than stakeholders' true positions. A limitation specific to this study is that a larger sample size, achieved by either a broader sampling frame or longer search period, would have increased the external validity of the findings. Further, as data on citations of stakeholders were collected as simple tallies, we could not investigate trends in representations of stakeholders over time. Future research could analyse media coverage subsequent to 2014 to examine how representations of stakeholders have evolved over time. Additionally, including other forms of media, including social media, may have added depth to understandings of the debate, particularly as e‐cigarette regulation seems to garner considerable engagement online. Despite these limitations, this study makes a valuable contribution to the literature on e‐cigarette regulation 16. By enabling comparison between the e‐cigarette debate and other tobacco control debates, our analysis may inform future advocacy in the United Kingdom and internationally. We suggest that public disagreement between tobacco control advocates may be harmful to shared health policy goals. However, rather than recommending against public debate, we would instead recommend emphasizing the substantial areas of consensus that exist.

Our data illustrate increased newspaper coverage of the e‐cigarette debate throughout 2013 and 2014, and comparison with data on UK newspaper coverage of other health legislation debates indicates that the e‐cigarette regulation debate occupied a similar number of articles as legislation to prohibit smoking in vehicles carrying children 35, but substantially fewer articles than proposed legislation to impose a minimum price per unit of alcohol 28. The sharp rise in reporting on e‐cigarette regulation in late 2014 suggests that the profile of the issue may have continued to rise in 2015.

We found that stakeholders supported e‐cigarette regulation much more frequently than they opposed it, suggesting that the overall tone of media representations of the debate was favourable to regulation. To an extent, this is foreseeable in a debate about potential regulation, as the presence of arguments for regulation is a prerequisite for the presence of opposing arguments. Governments, regulatory bodies, politicians and health charities were broadly aligned in support for regulation of purchasing age, regulation of marketing and regulating e‐cigarettes as medicine, and these measures were rarely opposed. Widespread support for the regulation of e‐cigarettes as medicine is probably founded on a perceived need to set the parameters of what e‐cigarettes and refills may be composed of, both for the protection of consumers, as highlighted in our data, and to ensure a uniform product for which further regulation can be designed. Purchasing age restrictions and regulation of marketing were frequently justified based on the need to protect children from harm, a rationale that industry actors have been unwilling to oppose in past tobacco control debates 36.

Prohibition of e‐cigarette use in enclosed public spaces was the key area of disagreement within and between the most vocal stakeholder groups. While comparable restrictions have been successful when applied to tobacco 37, evidence of the risks of second‐hand vaping is scarce 38, which may go some way to explaining the relative lack of enthusiasm for the measure. Advocates rationalized their support primarily by citing the risks of re‐normalizing smoking behaviours, which are more abstract and perhaps less persuasive than direct health risks. Additionally, those positioning e‐cigarettes as tobacco cessation tools portrayed the prohibition of their use in enclosed public spaces as counterproductive, as it would reduce tobacco smokers' incentives to adopt the (assumed) safer alternative. The disagreement exhibited by health charities on this issue illustrated the challenge they face in finding balance between the promise and threat of this disruptive technology.

Transnational tobacco companies had a low profile in the media debate in 2013 and 2014, indicating that TTCs’ attempted rehabilitation through engagement in harm reduction debates 39, 40 is not evident in UK newsprint coverage. Our data cannot explain the near‐absence of TTCs in the debate, but various explanations may be posited: TTCs may have chosen not to draw attention to their growing share of the e‐cigarette market; TTCs may be confident in their financial capacity to adapt to regulation (which could be prohibitively expensive for independent e‐cigarette companies); and TTCs may anticipate that regulation to standardize the nicotine content of e‐cigarettes will encourage profitable dual use of tobacco and e‐cigarettes. Additionally, TTCs may have decided not to reach conclusions ahead of the EU TPD in April 2014, in which case their profile may have risen subsequent to the sample period.

Tobacco control advocates have previously been unified largely around the regulatory measures they support 41, and coalitions and the promotion of unambiguous messages have been instrumental to successful advocacy 42, 43. Our analysis found that public health stakeholders demonstrated less unity in the e‐cigarette regulation debate than in previous tobacco control debates 41, and the two open letters addressed to the WHO by opposing groups of public health and medical experts 41 indicate that that disunity exists beyond media representations. The first letter advised the WHO to recognize the harm reduction potential of e‐cigarettes and reverse recommendations for regulation that would suppress their availability, while the second supported the WHO's existing precautionary stance on e‐cigarette regulation. Disagreement between public health stakeholders may be explained partially by the absence (prior to October 2014) of official international guidance on the issue, as WHO guidance has been found to have aided consensus‐building in past tobacco control advocacy 43, 44, 45, 46. Variations in stakeholders’ positions might also be explained by the incomplete evidence base concerning the harms and benefits of e‐cigarettes, differing starkly from the comprehensive evidence for the harms of tobacco.

Rather than solely attributing disagreements to limited evidence, we suggest that more fundamental barriers to agreement lie in the frameworks within which actors interpret and use that evidence base. Fairchild & Bayer 4 argue that differing assessments of e‐cigarettes stem from conflicting philosophical frameworks of public health: harm reduction and precaution. Harm reduction can be described as a pragmatic approach acknowledging that people will inevitably use drugs, and viewing risk minimization as a worthy public health goal, whereas precautionary approaches focus on the complete elimination of harmful habits, arguing that simply reducing harm is undesirable and cautioning against serving the interests of TTCs. The same evidence may be interpreted differently depending on the framework that is applied. Our analysis indicates that stakeholders using rationales commensurate with harm reduction (such as promoting e‐cigarettes as tobacco cessation aids or highlighting the lack of evidence of the risks of e‐cigarettes) tended to oppose the prohibition of e‐cigarette use in enclosed public spaces, while those using precautionary rationales (such as cautioning against the re‐normalization of smoking and the potential role of e‐cigarettes as gateways to tobacco) tended to favour comprehensive regulation. Stakeholders may model their stances towards regulation based on their pre‐existing adherence to a specific framework, but equally these frameworks may be used to post‐rationalize stances towards regulation.

Divisions between public health stakeholders exist within other tobacco control debates 47. For example, some forms of smokeless tobacco are promoted as tobacco cessation aids by some 48 and cautioned against by others, who highlight their carcinogenic content, the involvement of TTCs in their production and marketing and the inconclusive evidence of their effectiveness in tobacco cessation 49. Given that conflict within the tobacco control community is neither a new phenomenon nor one that relates exclusively to e‐cigarettes, confronting disagreement is of relevance beyond the e‐cigarette regulation debate. Industry actors with a history of opposing tobacco control legislation could exploit disagreement by characterizing tobacco control regulation as contested. This threat may incentivize public health advocates to develop common ground further and highlight existing agreement to present unified, unambiguous positions.

This paper contributes to the body of literature concerning mass media representations of public health policy and the dynamics of e‐cigarette regulation debates. While public health stakeholders are largely unified in support of e‐cigarette regulation, the disagreement that exists, concerning primarily the regulation of e‐cigarettes in public places, is evident in the public sphere. Given the persuasive power of presenting consistent messages in tobacco control debates, achieving consensus and agreeing on unambiguous advocacy positions on e‐cigarette regulation would probably increase the political effectiveness of the public health community. As public health stakeholders draw from divergent frameworks, reaching consensus is not simply a case of awaiting further research evidence, but one of negotiating shared values concerning how evidence is interpreted and presented. If critical engagement with public health frameworks is impractical, then a more pragmatic goal for the tobacco control community may be to refrain from commenting on contentious aspects of regulation in the public sphere to avoid gifting opponents of tobacco control the opportunity to exploit uncertainty, and focus instead on common areas of agreement. Ongoing debates about e‐cigarette regulation and other tobacco control issues in the United Kingdom and abroad may benefit from incorporating this approach.

### Declaration of interests

None.

## Supporting information


**Figure** **S1** Frequency of articles about e‐cigarette regulation by quarter

**Table** **S1** Summary of articles by region, genre and publication.

**Table** **S2** Frequency of citations of stakeholder categories.

**Table** **S3** Frequency of mentions of rationales for regulation by stakeholder category.

**Table** **S4** Frequency of mentions of rationales against regulation by stakeholder category.

## References

[1] Action on Smoking and Health . Electronic cigarettes; 2014.

[2] Bauld L. , De Andrade M., Angus K. E‐cigarette uptake and marketing: a report commissioned by Public Health England. Stirling, UK: Institute for Social Marketing, University of Stirling; 2014. Available at: https://www.gov.uk/government/publications/electronic‐cigarettes‐reports‐commissioned-by‐phe (accessed 1 April 2016).

[3] National Institute for Health and Care Excellence (NICE) . Tobacco: harm‐reduction approaches to smoking. NICE public health guidance 45. London: NICE; 2013.

[4] Fairchild A. L. , Bayer R. Smoke and fire over e‐cigarettes. Science 2015; 347: 375–6. 25613878 10.1126/science.1260761

[5] Bell K. , Keane H. Nicotine control: E‐cigarettes, smoking and addiction. Int J Drug Policy 2012; 23: 242–7. 22365155 10.1016/j.drugpo.2012.01.006

[6] West R. , Brown J. Electronic cigarettes: fact and faction. Br J Gen Pract 2014; 64: 442–3. 25179048 10.3399/bjgp14X681253 PMC4141591

[7] Hajek P. Electronic cigarettes have a potential for huge public health benefit. BMC Med 2014; 12: 225. 25491742 10.1186/s12916-014-0225-z PMC4260378

[8] Pisinger C. Why public health people are more worried than excited over e‐cigarettes. BMC Med 2014; 12: 226. 25488431 10.1186/s12916-014-0226-y PMC4260246

[9] McKee M. Electronic cigarettes: proceed with great caution. Int J Public Health 2014; 59: 683–5. 25135013 10.1007/s00038-014-0589-z

[10] MacCalman L. , Semple S. , Galea K. S. , Van Tongeren M. , Dempsey S. , Hilton S. , *et al.* The relationship between workers' self‐reported changes in health and their attitudes towards a workplace intervention: lessons from smoke‐free legislation across the UK hospitality industry. BMC Public Health 2012; 12: 324. 22551087 10.1186/1471-2458-12-324 PMC3407478

[11] Grana R. A. Electronic cigarettes: a new nicotine gateway? J Adolesc Health 2013; 52: 135–6. 23332475 10.1016/j.jadohealth.2012.11.007

[12] European Commission . EU directive 2014/40/EU of the European parliament and of the council. Off J Eur Union 2014; 57.

[13] Scottish Government . Scottish Government Response to Consultations on Electronic Cigarettes Tobacco: Wilful Neglect/Ill Treatment and Duty of Candour. Edinburgh: Scottish Government; 2015.

[14] Department of Health . Government response to the consultation on an age of sale for nicotine inhaling products London: Department of Health; 2015: 6–9.

[15] Scottish Parliament . Health (Tobacco, Nicotine etc. and Care) (Scotland) Bill.Edinburgh: Scottish Parliament; 2015.

[16] Rooke C. , Amos A. News media representations of electronic cigarettes: an analysis of newspaper coverage in the UK and Scotland . Tob Control 2014; 23: 507–12. 23884011 10.1136/tobaccocontrol-2013-051043

[17] Harrabin R. , Coote A. , Allen J. Health in the news: risk, reporting and media influence. London: King's Fund London; 2003.

[18] Burstein P. The impact of public opinion on public policy: a review and an agenda. Polit Res Q 2003; 56: 29–40.

[19] Kitzinger J. Framing Abuse: Media Influence and Public Understanding of Sexual Violence Against Children. London: Pluto Press; 2004.

[20] McCombs M. A look at agenda‐setting: past, present and future. J Stud 2005; 6: 543–57.

[21] Scheufele D. A. , Tewksbury D. Framing, agenda setting, and priming: the evolution of three media effects models. J Commun 2007; 57: 9–20.

[22] Entman R. M. Framing bias: media in the distribution of power. J Commun 2007; 57: 163–73.

[23] Kahneman D. , Tversky A. Choices, values, and frames. Am Psychol 1984; 39: 341.

[24] Callaghan K. , Schnell F. Assessing the democratic debate: how the news media frame elite policy discourse. Polit Commun 2001; 18: 183–213.

[25] Scottish Government . Creating a tobacco‐free generation: a tobacco control strategy for Scotland. Edinburgh: Scottish Government; 2013.

[26] Department of Health and Welsh Government . Age of sale for nicotine inhaling products: consultation document. 2014.

[27] Scottish Government . A Consultation on Electronic Cigarettes and Strengthening Tobacco Control in Scotland. Edinburgh: Scottish Government; 2014.

[28] Patterson C. , Katikireddi S. V. , Wood K. , Hilton S. Representations of minimum unit pricing for alcohol in UK newspapers: a case study of a public health policy debate. J Public Health 2015; 37: 40–9. 10.1093/pubmed/fdu078 PMC4340327 25312002

[29] Hilton S. , Wood K. , Bain J. , Patterson C. , Duffy S. , Semple S. Newsprint coverage of smoking in cars carrying children: a case study of public and scientific opinion driving the policy debate. BMC Public Health 2014; 14: 1116. 25351408 10.1186/1471-2458-14-1116 PMC4230725

[30] Hilton S. , Patterson C. , Teyhan A. Escalating coverage of obesity in UK newspapers: the evolution and framing of the ‘obesity epidemic’ from 1996 to 2010. Obesity 2012; 20: 1688–95. 22318314 10.1038/oby.2012.27 PMC3408646

[31] National Readership Survey . NRS Readership Estimates—Newspapers and Supplements. London: National Readership Survey; 2015.

[32] Aronson J. A pragmatic view of thematic analysis. Qual Rep 1995; 2: 1–3.

[33] Electronic Cigarette Industry Trade Association (ECITA) . ECITA Members. Neath, UK: ECITA; 2015.

[34] Morley C. P. The politics of tobacco regulation. Eur J Pub Health 2015; 25: 186. 25818490 10.1093/eurpub/ckv004

[35] Patterson C. , Semple S. , Wood K. , Duffy S. , Hilton S. A quantitative content analysis of UK newsprint coverage of proposed legislation to prohibit smoking in private vehicles carrying children. BMC Public Health 2015; 15: 760. 26253515 10.1186/s12889-015-2110-x PMC4529703

[36] Freeman B. , Chapman S. , Storey P. Banning smoking in cars carrying children: an analytical history of a public health advocacy campaign. Aust NZ J Public Health 2008; 32: 60–5. 10.1111/j.1753-6405.2008.00167.x 18290915

[37] Menzies D. , Nair A. , Williamson P. A. , Schembri S. , Al‐Khairalla M. Z. H. , Barnes M. , *et al.* Respiratory symptoms, pulmonary function, and markers of inflammation among bar workers before and after a legislative ban on smoking in public places. JAMA 2006; 296: 1742–8. 17032987 10.1001/jama.296.14.1742

[38] Hajek P. , Etter J.‐F. , Benowitz N. , Eissenberg T. , McRobbie H. Electronic cigarettes: review of use, content, safety, effects on smokers and potential for harm and benefit. Addiction 2014; 109: 1801–10. 25078252 10.1111/add.12659 PMC4487785

[39] Peeters S. , Gilmore A. B. Transnational tobacco company interests in smokeless tobacco in europe: analysis of internal industry documents and contemporary industry materials. PLOS Med 2013; 10: e1001506. 10.1371/journal.pmed.1001506 PMC3769209 24058299

[40] De Andrade M. , Hastings G. Tobacco harm reduction and nicotine containing products: research priorities and policy directions. London: Cancer Research UK; 2013.

[41] Künzli N. To e‐smoke or not to e‐smoke: is that a question? Int J Public Health 2014; 59: 679–80. 25194566 10.1007/s00038-014-0598-y

[42] Princen S. Advocacy coalitions and the internationalization of public health policies. J Publ Pol 2007; 27: 13–33.

[43] Weishaar H. , Collin J. , Amos A. Tobacco control and health advocacy in the European Union: understanding effective coalition‐building. Nicotine Tob Res 2015; 18(2): 122–129. 25634938 10.1093/ntr/ntv016 PMC4710205

[44] World Health Organization (WHO) . WHO Framework Convention on Tobacco Control. Geneva: WHO; 2003.

[45] Weishaar H. , Amos A. , Collin J. Best of enemies: using social network analysis to explore a policy network in European smoke‐free policy. Soc Sci Med 2015; 133: 85–92. 25863723 10.1016/j.socscimed.2015.03.045

[46] Mamudu H. M. , Glantz S. A. Civil society and the negotiation of the framework convention on tobacco control. Glob Public Health 2009; 4: 150–68. 19333806 10.1080/17441690802095355 PMC2664518

[47] O'Connor R. J. Non‐cigarette tobacco products: what have we learnt and where are we headed? Tob Control 2012; 21: 181–90. 22345243 10.1136/tobaccocontrol-2011-050281 PMC3716250

[48] Foulds J. , Kozlowski L. Snus—what should the public‐health response be? Lancet 2007; 369: 1976–8. 17498796 10.1016/S0140-6736(07)60679-5

[49] McKee M. , Gilmore A. Swedish snus for tobacco harm reduction. Lancet 2007; 370: 1206. 17920913 10.1016/S0140-6736(07)61530-X

