# Supplementary figures and images for: Who thinks what about e‐cigarette regulation? A content analysis of UK newspapers
Source: Addiction. 2016 Mar 11;111(7):1267–74. doi: 10.1111/add.13320 (PMC4982091; doi:10.1111/add.13320)

**Figure S1.** Frequency of articles about e-cigarette regulation by quarter


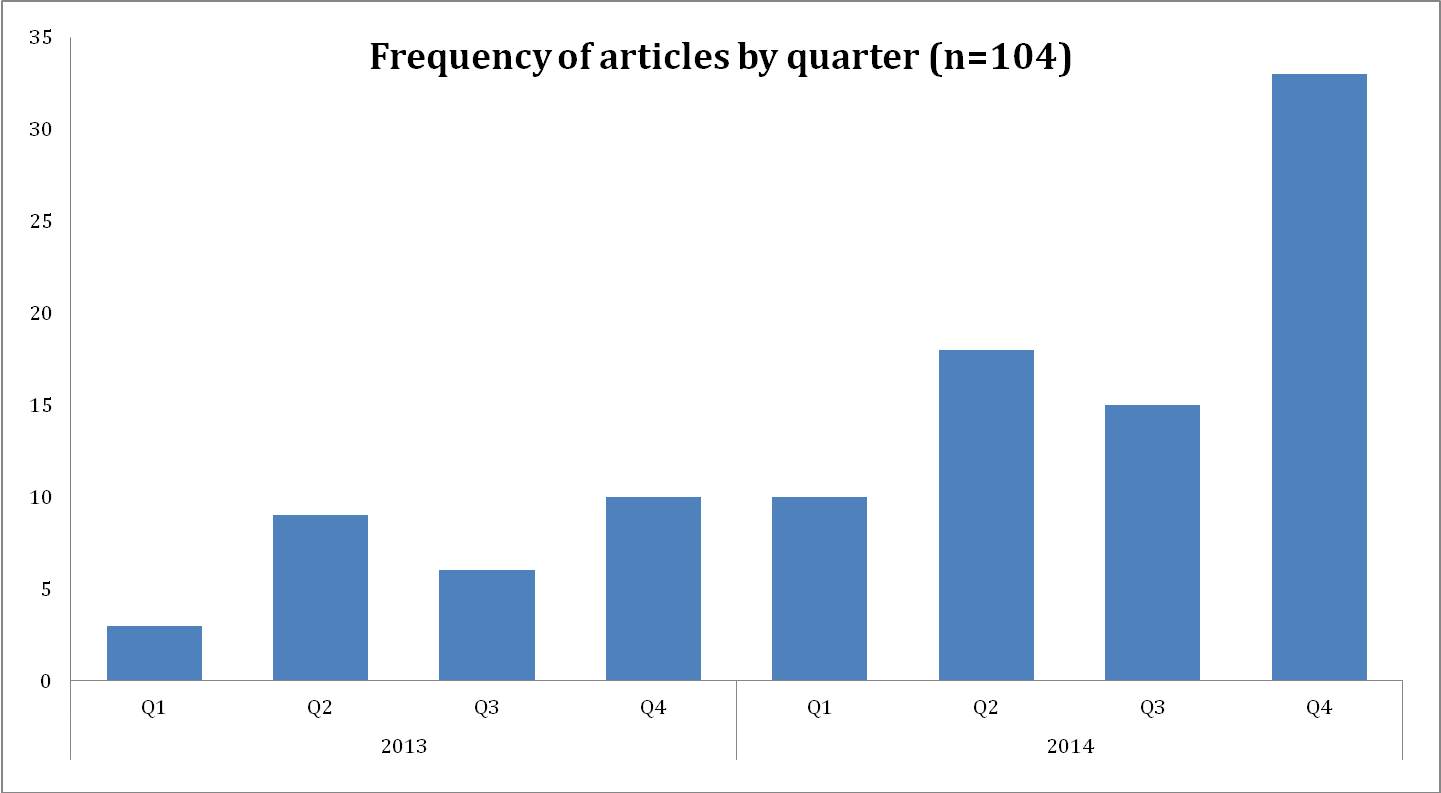

Supplement: Supplementary file 1 — Figure S1 Frequency of articles about e‐cigarette regulation by quarter [file ADD-111-1267-s001.docx]
