# Supplementary material for: Who thinks what about e‐cigarette regulation? A content analysis of UK newspapers
Source: Addiction. 2016 Mar 11;111(7):1267–74. doi: 10.1111/add.13320 (PMC4982091; doi:10.1111/add.13320)
Supplement: Supplementary file 2 — Table S1 Summary of articles by region, genre and publication. [file ADD-111-1267-s002.docx]

**Table S1. Summary of articles by region, genre and publication**

| **Region** | **Genre** | **n** | **%** | **Publication** | **n** | **%** |
| --- | --- | --- | --- | --- | --- | --- |
| UK (n=76) | Tabloid | 23 | 22.1 | The Sun | 14 | 13.5 |
|  |  |  |  | Mirror & Sunday Mirror | 9 | 8.7 |
|  | Middle-market tabloid | 21 | 20.2 | Daily Mail & Mail on Sunday | 17 | 16.3 |
|  |  |  |  | Daily Express & Sunday Express | 4 | 3.8 |
|  | Quality | 32 | 30.8 | Guardian & Observer | 16 | 15.4 |
|  |  |  |  | Independent & Independent on Sunday | 5 | 4.8 |
|  |  |  |  | The Times & Sunday Times | 2 | 1.9 |
|  |  |  |  | Daily Telegraph & Sunday Telegraph | 9 | 8.7 |
| Scotland (n=28) | Tabloid | 5 | 4.8 | Daily Record & Sunday Mail | 5 | 4.8 |
|  | Quality | 23 | 22.1 | Scotsman & Scotland on Sunday | 13 | 12.5 |
|  |  |  |  | Herald & Sunday Herald | 10 | 9.6 |
|  |  |  |  | Total: | 104 | 100 |
