# Supplementary material for: Who thinks what about e‐cigarette regulation? A content analysis of UK newspapers
Source: Addiction. 2016 Mar 11;111(7):1267–74. doi: 10.1111/add.13320 (PMC4982091; doi:10.1111/add.13320)
Supplement: Supplementary file 3 — Table S2 Frequency of citations of stakeholder categories. [file ADD-111-1267-s003.docx]

**Table S2.** Frequency of citations of stakeholder categories

| **Stakeholder category** | **Citations** | **Statements supporting regulation** | **Statements opposing regulation** |
| --- | --- | --- | --- |
| **Government & regulatory bodies** | **50** | **66** | **1** |
| World Health Organisation | 14 | 24 | 0 |
| Scottish Government | 12 | 12 | 0 |
| United Kingdom (inc. DH, MHRA, PHE) | 10 | 16 | 1 |
| European (inc. EU, EC, EP) | 6 | 6 | 0 |
| Welsh | 3 | 2 | 0 |
| German | 1 | 0 | 0 |
| United States Federal Government | 1 | 1 | 0 |
| United States Local Government | 1 | 0 | 0 |
| French Government | 1 | 3 | 0 |
| Queensland Australia Government | 1 | 2 | 0 |
| **Politicians** | **20** | **7** | **9** |
| Conservative Party | 6 | 1 | 4 |
| Scottish National Party | 5 | 2 | 0 |
| Welsh Labour | 3 | 3 | 0 |
| Liberal Democrats | 2 | 0 | 2 |
| United Kingdom Independence Party | 2 | 0 | 2 |
| Scottish Conservative Party | 1 | 0 | 1 |
| Green Party | 1 | 1 | 0 |
| **Health charities** | **18** | **12** | **8** |
| ASH | 8 | 5 | 4 |
| ASH Scotland | 8 | 5 | 4 |
| Northern Ireland Chest Heart & Stroke | 1 | 1 | 0 |
| Royal Environmental Health Institute of Scotland | 1 | 1 | 0 |
| **E-cigarette industry** | **15** | **7** | **8** |
| Electronic Cigarette Industry Trade Association | 5 | 4 | 4 |
| E-lites | 4 | 0 | 1 |
| Skycig | 1 | 2 | 0 |
| Gamucci | 1 | 0 | 0 |
| JAC Vapour | 1 | 1 | 0 |
| Socialites | 1 | 0 | 1 |
| Totally Wicked | 1 | 0 | 1 |
| Vapestick | 1 | 0 | 1 |
| **Bodies representing health professionals** | **12** | **15** | **0** |
| British Medical Association | 11 | 14 | 0 |
| Royal Society for Public Health | 1 | 1 | 0 |
| **Academics** | **8** | **3** | **4** |
| University College London | 3 | 0 | 2 |
| United Kingdom Centre for Tobacco Control Studies | 3 | 1 | 1 |
| University of Edinburgh | 1 | 1 | 0 |
| Centre for Drug Misuse Research | 1 | 1 | 1 |
| **Tobacco lobbyists** | **2** | **0** | **4** |
| FOREST | 2 | 0 | 4 |
| **Others** | **2** | **1** | **1** |
| Jodie Marsh (celebrity) | 1 | 0 | 1 |
| 60 millions de consommateurs (consumer magazine) | 1 | 1 | 0 |
| **Total:** | **127** | **111** | **35** |

Note: Some citations contained multiple arguments and some citations contained no clear arguments. Therefore, the total frequency of arguments for and against regulation (n=146) does not equal the total frequency of citations (n=127).
