# Supplementary material for: Who thinks what about e‐cigarette regulation? A content analysis of UK newspapers
Source: Addiction. 2016 Mar 11;111(7):1267–74. doi: 10.1111/add.13320 (PMC4982091; doi:10.1111/add.13320)
Supplement: Supplementary file 4 — Table S3 Frequency of mentions of rationales for regulation by stakeholder category. [file ADD-111-1267-s004.docx]

**Table S3.** Frequency of mentions of rationales for regulation by stakeholder category

|  | **Government & regulatory bodies** | **Health charities** | **Bodies representing health professionals** | **Politicians** | **E-cigarette industry** | **Academics** | **Others** | **Total** |
| --- | --- | --- | --- | --- | --- | --- | --- | --- |
| **Rationales for regulation** |  |  |  |  |  |  |  |  |
| Protect children and young people | 20 | 9 | 5 | 3 | 5 | 1 | 1 | **44** |
| Need to ensure safety and quality | 13 | 4 | 2 | 1 | 2 | 1 | 1 | **24** |
| Prevent re-normalisation of smoking | 8 | 1 | 2 | 2 | 0 | 0 | 0 | **13** |
| E-cigarettes are a gateway to tobacco | 1 | 1 | 0 | 3 | 0 | 1 | 0 | **6** |
| Limit second-hand exposure to vapour | 4 | 0 | 1 | 0 | 0 | 0 | 0 | **5** |
| Protect public health | 3 | 0 | 0 | 0 | 0 | 0 | 0 | **3** |
| **Total:** | **49** | **15** | **10** | **9** | **7** | **3** | **2** | **95** |
