# Supplementary material for: Who thinks what about e‐cigarette regulation? A content analysis of UK newspapers
Source: Addiction. 2016 Mar 11;111(7):1267–74. doi: 10.1111/add.13320 (PMC4982091; doi:10.1111/add.13320)
Supplement: Supplementary file 5 — Table S4 Frequency of mentions of rationales against regulation by stakeholder category. [file ADD-111-1267-s005.docx]

**Table S4.** Frequency of mentions of rationales against regulation by stakeholder category

|  | **E-cigarette industry** | **Politicians** | **Health charities** | **Academics** | **Others** | **Smoker's rights groups** | **Total** |
| --- | --- | --- | --- | --- | --- | --- | --- |
| **Rationales against regulation** |  |  |  |  |  |  |  |
| E-cigarettes are a tobacco cessation aid | 2 | 5 | 2 | 1 | 1 | 0 | **11** |
| Lack of evidence that e-cigarettes are unsafe | 5 | 2 | 2 | 0 | 1 | 1 | **11** |
| Regulation would push consumers back to tobacco | 0 | 2 | 1 | 1 | 0 | 0 | **4** |
| Regulation would benefit the tobacco industry | 2 | 0 | 0 | 0 | 0 | 0 | **2** |
| E-cigarettes are not medicine | 1 | 0 | 0 | 0 | 0 | 0 | **1** |
| Regulation would increase prices | 1 | 0 | 0 | 0 | 0 | 0 | **1** |
| **Total:** | **11** | **9** | **5** | **2** | **2** | **1** | **30** |
